# Stratified analyses of genome wide association study data reveal haplotypes for a candidate gene on chromosome 2 (*KIAA1211L*) is associated with opioid use in patients of Arabian descent

**DOI:** 10.1186/s12888-019-2425-8

**Published:** 2020-01-31

**Authors:** Hiba Alblooshi, Habiba Al Safar, Ahmed El Kashef, Hamad Al Ghaferi, Mansour Shawky, Gary K. Hulse, Guan K. Tay

**Affiliations:** 1 Division of Psychiatry, the University of Western Australia, Crawley, Western Australia Australia; 2 School of Human Science, The University of Western Australia, Crawley, Western Australia Australia; 3 College of Medicine and Health Science, The United Arab Emirates University, Al Ain, United Arab Emirates; 4 Center of Biotechnology, Khalifa University of Science and Technology, Abu Dhabi, United Arab Emirates; 5 Department of Biomedical Engineering, Khalifa University of Science and Technology, Abu Dhabi, United Arab Emirates; 6 National Rehabilitation Center, Abu Dhabi, United Arab Emirates; 7 School of Health and Medical Science, Edith Cowan University, Joondalup, Western Australia Australia

**Keywords:** Opioid use disorder, GWAS, stratification, Haplotypes, *KIAA1211L*

## Abstract

**Background:**

Genome Wide Association Studies (GWAS) have been conducted to identify genes and pathways involved in development of opioid use disorder. This study extends the first GWAS of substance use disorder (SUD) patients from the United Arab Emirates (UAE) by stratifying the study group based on opioid use, which is the most common substance of use in this cohort.

**Methods:**

The GWAS cohort consisted of 512 (262 case, 250 controls) male participants from the UAE. The samples were genotyped using the Illumina Omni5 Exome system. Data was stratified according to opioid use using PLINK. Haplotype analysis was conducted using Haploview 4.2.

**Results:**

Two main associations were identified in this study. Firstly, two SNPs on chromosome 7 were associated with opioid use disorder, rs118129027 (*p*-value = 1.23 × 10 ^− 8^) and rs74477937 (*p*-value = 1.48 × 10 ^− 8^). This has been reported in Alblooshi et al. (Am J Med Genet B Neuropsychiatr Genet 180(1):68–79, 2019). Secondly, haplotypes on chromosome 2 which mapped to the *KIAA1211L* locus were identified in association with opioid use. Five SNPs in high linkage disequilibrium (LD) (rs2280142, rs6542837, rs12712037, rs10175560, rs11900524) were arranged into haplotypes. Two haplotypes GAGCG and AGTTA were associated with opioid use disorders (*p*-value 3.26 × 10^− 8^ and 7.16 × 10^− 7^, respectively).

**Conclusion:**

This is the first GWAS to identify candidate genes associated with opioid use disorder in participants from the UAE. The lack of other genetic data of Arabian descent opioid use patients has hindered replication of the findings. Nevertheless, the outcomes implicate new pathways in opioid use disorder that requires further research to assess the role of the identified genes in the development of opioid use disorder.

## Background

Epidemiological data from the 2010 Global Burden of Diseases Health Measurement Survey estimated that some 15.5 Million people across the globe were afflicted with opioid use disorder [1]. As a measure of the chronic nature of the problem, the use of opioids significantly rose in the United States of America (USA) to epidemic proportions with a dramatic increase of 78% in 2013 [2]. Opioids of choice included heroin, as well as the substances morphine, fentanyl, oxycodone and tramadol which are widely used as prescribed analgesics for surgery [3]. The rise in the use of prescribed opioids for non-medical purposes has been reviewed in many countries [4]. The USA is considered the epicentre of the world’s prescription drugs problem, where a 10 to 14-fold increase in prescription medication has been estimated [5, 6]. The consumption of prescription medication for non-medical use resulting in harmful effects in 2010, was highest in the USA at 47,809 (consumption level per capita), followed by Canada (26,380), the United Kingdom (UK) (10,297) and Australia (12,654) [7]. In addition, there was a massive increase of 430% in the number of treatment admission for prescription medication misuse between 1999 to 2009 in the USA [6]. Even though, there been as a dramatic increase in the prescription medication misuse in Europe, it is not as prevalent in the UK in comparison to the USA [8, 9]. This is due to the strict regulation and legislations that control the use of prescription medication in the UK through the implementation of an electronic prescription system [4]. However, around 30% of patients with prescribed medication in the UK tend to sell or swap their allocation with other medications that contribute to the problem [10]. In North Africa and Middle East region, an estimated 1.37 million patients reported the use of opioids in 2010 [1]. In the first retrospective study of substance use disorder (SUD) in the United Arab Emirates (UAE) from 2002 to 2011, opioids (heroin = 16.3%) were the second most common substance of use [11].

This pattern of prescription opioid use in the young (below the age of 30 years old) users from the UAE was recently described in Alblooshi et al (2016) [12]. The use of prescription opioids such as Tramadol rose by an estimated 67.2% in users below 30 years of age [12, 13]. This cohort was used in this study.

The vulnerability to substance use and treatment responses is partly affected by genetic factors [14–16] . The Identification of genes that contribute to the development of the disease can improve treatment outcomes of SUD. Opioid receptors (*OPRM1*, *OPRD1* and *OPRK1*) have been extensively studied in addiction due to their involvement in the reward pathways. The μ-opioid receptor (*OPRM1*) has been the main focus in opioid use disorder. Various Genome Wide Association Studies (GWAS) [17–19] have looked into the genetic factors contributing to opioid use disorder. However, inconsistent genetic associations have been reported, suggesting that there are other systems involved in the pathogenesis of the opioid use disorder [20]. In the first GWAS study by Gelenter et al (2014) [17] the *KCNG2* (rs62103177) gene on chromosome 18 was implicated. The association was mapped to a Calcium and Potassium pathway, a novel risk pathway that provided a new direction for therapeutic and preventative strategies [17]. Nelson et al (2015) [21] subsequently reported an association of the cornichon family AMPA receptor auxiliary protein 3 gene (*CNIH3*) with heroin use disorder. This finding implicated the involvement of the glutamate system in the pathophysiology of opioid use disorder. To date, a number of genes, including *LOC647946*, *FAM53B*, *CRYGS* which encode proteins involved in different biochemical pathways have been reported. Presently, no definitive mechanism has been uncovered to explain the underlining pathophysiology of the opioid use disorder.

In many polygenic diseases, ethnic specific genetic variations have been described. In opioid use disorder, various GWA studies have been performed and reported in European American, African American [17, 22] and Australian population [21]. However, none have been performed on an Arab population. In this study, the first GWAS of SUD in a population of Arabian descent was conducted. The subjects were opioid users as this substance class was the most common substance of use at 80.4% [12] of the cohort studied. Three novel variants on chromosome 7 were identified and discussed in Alblooshi et al [23]. In this report, associations with haplotypes on chromosome 2 around the *KIAA1211L* locus are presented.

## Methods

### Participants

The GWAS discovery samples consisted of 262 male participants from the UAE. Cases included 250 male patients from the UAE National Rehabilitation Centre (NRC). All cases were diagnosed with SUD based on Diagnostic and Statistical Manual-5 (DSM-5) criteria. However, cases were not assessed for other psychiatric disorders at the time of recruitment. Controls with no prior history of SUD were retrieved from the Emirates Family Registry (EFR) [24] as a control group. However, other diseases were included in the control group selection criteria such as diabetes, cardiovascular diseases, dyslipidaemia, etcetera. The details of the cohort have been previously summarised in Alblooshi, et al (2016) [12] including the demographic characteristic and the type of substances used. The cohort was stratified based according to the common substance of use, which was opioids.

The study was conducted in accordance with standards set by the World Medical of Helsinki [25]. The ethics committee of the National Rehabilitation Centre (NRC) in Abu Dhabi, UAE reviewed and approved the study. Reciprocal approvals were received from the ethics committee of the University of Western Australia (RA/4/1/6715). Only participants who signed written informed consent were studied.

### Genotyping and quality control

Saliva samples were collected using the Oragene saliva kit (DNA Genoteck, Ottawa, Ontario, Canada). Genomic DNA was extracted using laboratory protocol for manual extraction as recommended by Genoteck. Each sample was processed for quantification using standard gel electrophoresis and Tecan NanoQuant Plate™ (*Infinite 200 Pro*) (Tecan Group, Männedorf, Switzerland). After extracting genomic DNA, the samples were genotyped using the Illumina Omni5 Exome (Illumina, San Diego, California), which contained 4.6 million Single Nucleotide Polymorphism (SNPs). Standard protocols as recommended by Illumina were used for hybridizing samples on the chip and scanning on the Illumina HiScan platform.

Quality Control (QC) step was applied on markers and individuals using PLINK [26]. The markers were filtered based on a genotype call rate < 99.6%; Minor Allele Frequency (MAF) < 0.05; significant deviation from Hardy Weinberg Equilibrium (*p*-value < 10^− 6^) and large difference (*p*-value < 0.00001) in missing data rate between cases and controls. The individuals were removed from the analysis where there was substantial missing data (> 90%); when whole genome heterozygosity was greater than three standard deviation from the mean; estimated proportion of “identity by descent” (IBD) sharing with another sample > 0.1, or gender discordant (based on X chromosomal heterozygosity) > 0.2. Multi-dimensional scaling (MDS) was carried out to identify population outliers. After filtering for individuals, 199 cases with opioid use disorder and 262 controls were retained final analysis.

### Statistical analysis

For the data stratified according to opioid use disorder, a total of 1,879,623 SNPs and 452 (Case:199 and Control:253) passed QC. These were included in the study analysis. The GWAS association test was preformed using Factored Spectrally Transformed Linear Mixed Model “FaST-LMM” [27]. Quantile-Quantile (QQ) and Manhattan plot were illustrated using R statistical package (R core team, Vienna, Austria). The GWAS significant level was set at 5.00 × 10^− 8^ [28]. The Regional Manhattan plot showing the SNP positions of interest were plotted using zoom locus webserver [29]. PLINK [26] was used for stratification of common substance of use. Haploview 4.2 [30] was used for haplotype and linkage disequilibrium (LD) analyses.

## Results

**Fig. 1:**
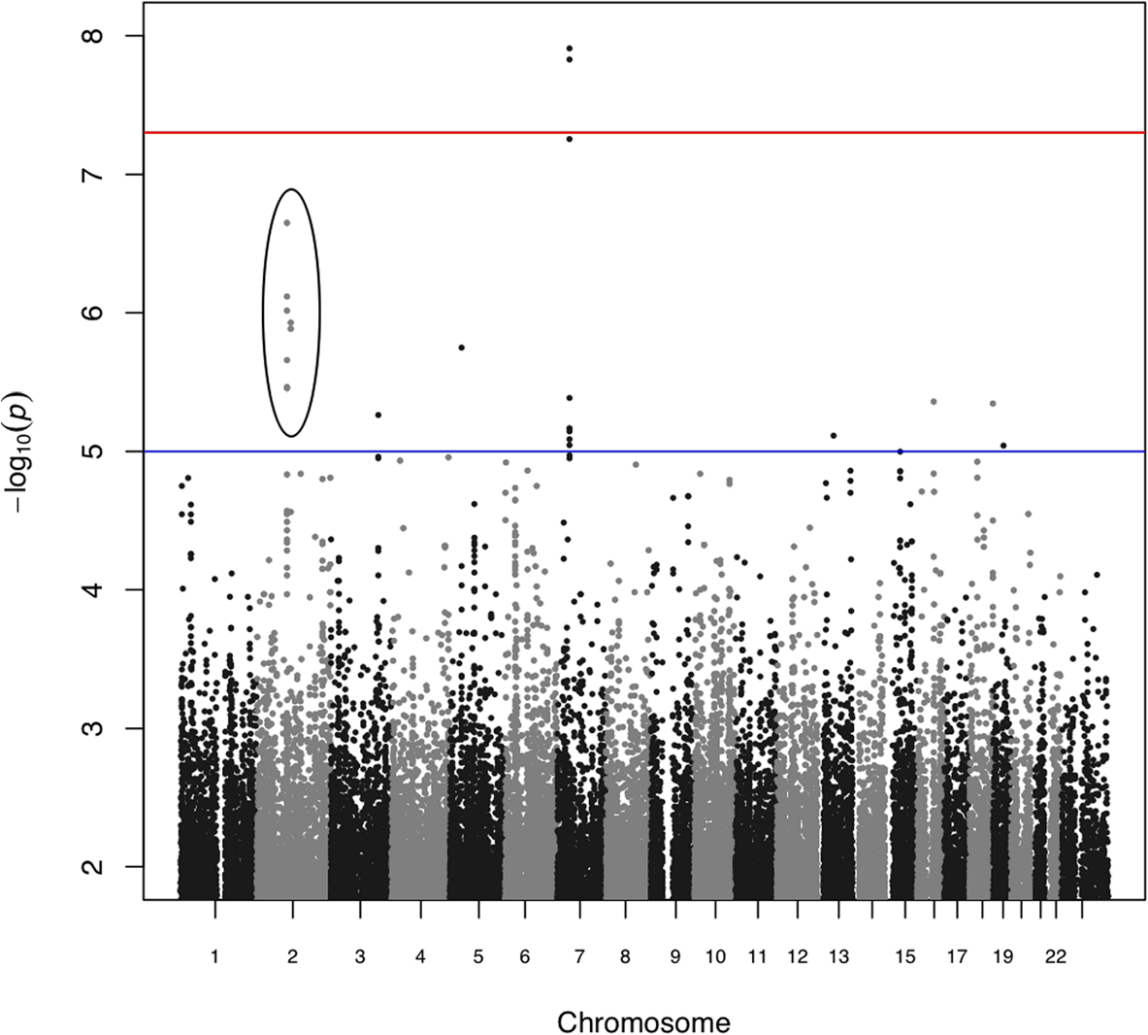
Manhattan plot shows *p*-value for stratified opioid users versus normal controls using FaST-LMM analysis. The X-axis represents the SNP markers in each chromosome and Y-axis represent the negative logarithm of *P*-values. The highest *p-*values were observed for rs118129027(*p-*value = 1.236 X 10 ^− 8^) and rs74477937 (*p-*value = 1.485 X 10 ^− 8^) on chromosome 7. The circle indicates a candidate haplotype that associated with opioid use disorder

In this report, analysis from a GWA study of SUD patients using opioids is presented. Fig. 1 shows the stratified GWAS Manhattan plot for opioid use disorder. In the analysis, two SNPs reached significant association with *p*-values of 1.23 × 10 ^− 8^ and 1.48 × 10 ^− 8^ for rs118129027 and rs74477937 respectively. These SNPs were localized to chromosome 7. Another SNP rs78707086 on chromosome 7 failed to reach the set GWAS significance level (*p*-value = 5.00 × 10^− 8^) but was suggestive of an association. Nevertheless, this third SNP was in LD with rs118129027 and rs74477937. The relevance of these three SNPs in SUD has been reported elsewhere [23].

A second association is described in this paper. Between the GWAS level of significance (5.00 × 10^− 8^) and the suggestive level (1.00 × 10^− 5^), seven SNPs on chromosome 2 formed a cluster with five of these SNPs in LD (Fig. 1). This suggested the possibility of candidate locus on chromosome 2 associated with opioid use disorder.

**Fig. 2:**
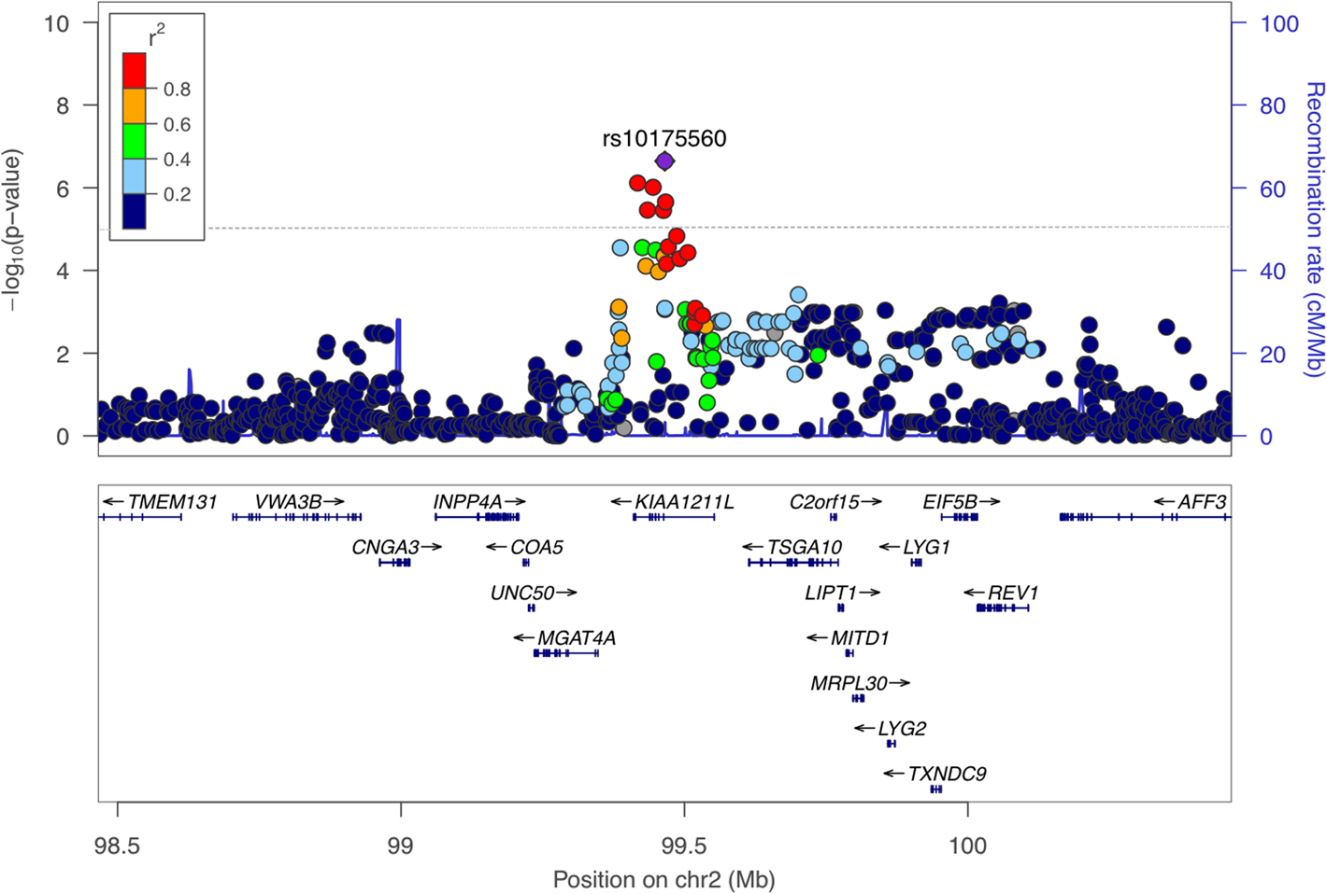
Regional Manhattan plot of the chromosome 2q11.2 region showing the FaSTLMM analysis of the opioid use disorder patients compared to controls. The LD heat map is based on the hg19/1000genomes NOV/2014 reference set. The SNPs are colour coded according to r^2^ measured based on the pairwise LD with the index SNP rs10175560 (*p*-value = 2.24 × 10 ^− 7^) shown in purple. Two other SNPs mapped to the *KIAA1211L* gene clustered in high LD indicating a possible candidate locus for opioid use disorder

The cluster of SNPs circled in Fig. 1 highlight the nucleotide positions that have risen above the suggestive GWAS confidence level. A regional manhattan plot (Fig. 2) was generated around the location of these SNPs, partly to assess Linkage Disequilibrium (LD) between the SNPs. The recombination rates are shown in centimorgans (cM) per megabase (Mb). Each SNP is represented as a circle with the y-axis shown as -log_10_ *p*-value. The SNP with the highest *p*-value (rs10175560 with a *p*-value = 2.24 × 10^− 7^) was selected as the index SNP (purple diamond in Fig. 2). Linkage disequilibrium (LD) values with other SNPs in the vicinity of this index were estimated in r^2^ values and shown in different colours. The LD correlation was estimated using the data from the 1000 Genome project [29]. The highest LD is shown in red were for SNPs with r^2^ > 0.08. SNPs with no LD data were represented by grey circles. Genes within the vicinity of the SNP of interest are provided. In this figure, the suggestive SNPs mapped to a region coinciding with a locus known as *KIAA1211L.* In addition, Fig. 2 illustrated strong LD that suggested further haplotype analysis for an association and improved evidence for candidate genes.

**Fig. 3:**
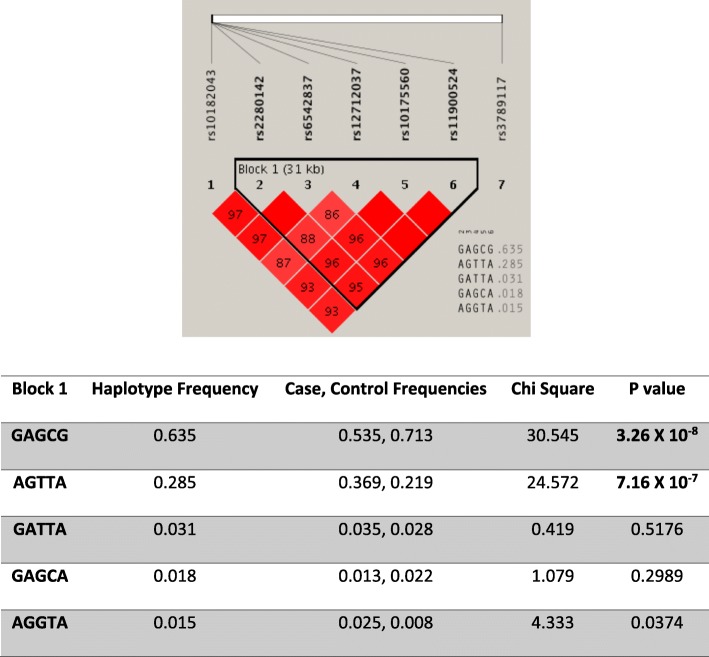
The state of the LD between the SNPs located above the GWAS suggestive line 1 X 10 ^−5^on chromosome 2, based on opioid use disorder patients compared to controls from the UAE population. Block 1 includes 5 SNPs mapped to the *KIAA1211L* gene that are significantly associated with opioid use. The numbers in the square represent the percentage of the r^2^ value calculated from the genotype data of the SNPs. The haplotype variations, frequencies and P-values illustrated with the GAGCG (*p*-value = 3.26 X 10^− 8^) haplotype the most significant

The SNPs on chromosome 2 that were above the suggestive line of 1.00 X 10 ^− 5^ determined by this GWA study are shown in Fig. 3. The data was not adjusted for other psychiatric disorder; hence the association level might be altered. Five out of seven SNPs on chromosome 2 above this suggestive line were in LD (rs2280142, rs6542837, rs12712037, rs10175560, rs11900524). These 7 SNPs were organized into haplotypes, with block 1 including the five SNPs: rs2280142, rs6542837, rs12712037, rs10175560, and rs11900524. Two haplotypes GAGCG and AGTTA were associated with opioid use disorders at *p*-values of 3.26 × 10^− 8^ and 7.16 × 10^− 7^, respectively.

**Fig. 4:**
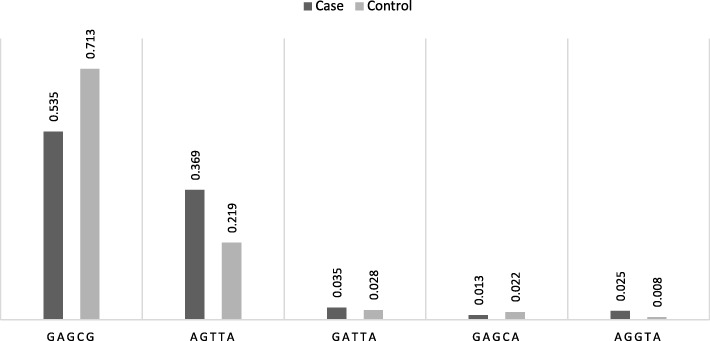
The distribution of the haplotypes in block 1 that include 5 SNPs between opioid users and controls. Haplotype 1 (GAGCG) the most statistically significant with higher frequency (0.713) in control group. Haplotype 2 (AGTTA) higher in cases (0.369) than control (0.219)

Fig. 4 provides a summary of the 5-point haplotype frequencies for block 1 in opioid users and controls. The frequency of haplotype 1 (GAGCG) at 0.713 was higher in the control group relative to the cases. In contrast, the frequency of haplotype 2 (AGTTA) was higher in cases (0.369) than in the control group (0.219).

The analysis was extended to include the chromosome 2 SNPs above the *p*-value 1 × 10 ^− 4^. Linkage Disequilibrium data of 31 SNPs is shown in Additional file 1: Figure. S1. Six haplotype blocks were constructed using these 31 SNPs. Block 1 and 2 mapped to the chromosomal region that coincided with *KIAA1211L*. Additional file 2: Table S1 also provides a summary of the haplotype distribution, the frequencies of each haplotype, and *p*-value of the cases and control groups.

**Fig. 5:**
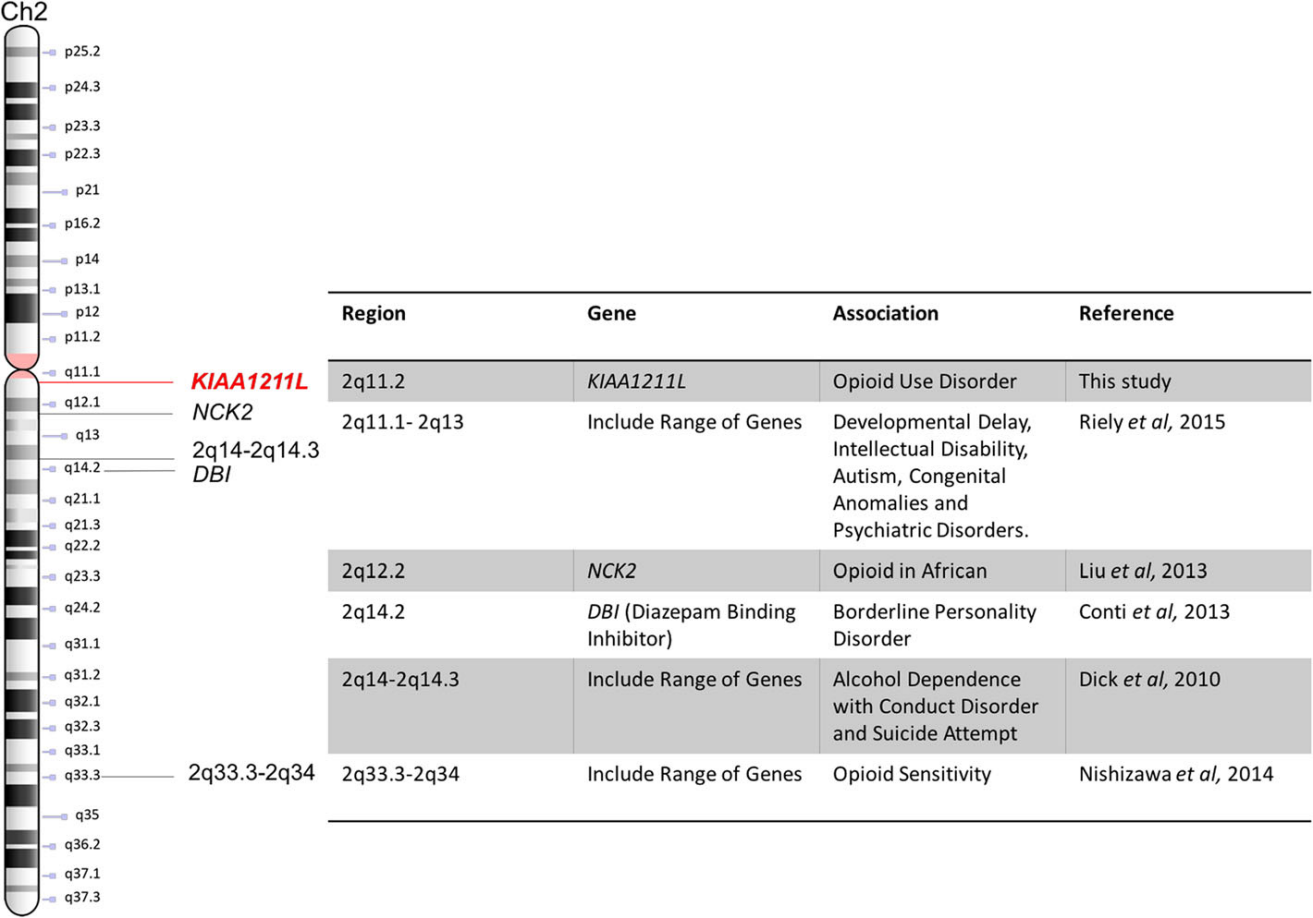
Published associations for chromosome 2q regions from 2q11 to 2q34 and SUD as well as other psychiatric disorders

A number of locations on chromosome 2 was shown to be associated with substance use and other psychiatry disorders. Fig. 5 summaries these association including the *KIAA1211L* loci identified in this study.

## Discussion

This study is an extension of a GWA study, the first of its kind, involving SUD patients in the UAE population. In the initial GWAS, three SNPs: rs118129027, rs74477937 and rs78707086 were below the GWAS significance level but highly suggestive of an association. These mapped to a region on chromosome 7, which coincides with the *YAE1D1* locus. Separately, the function of this gene was discussed as a possible candidate locus for substance use disorder [23]. For this study, the initial GWAS was stratified based on the most common substance of use, which were opioids. These are the most common substance of use in the cohort and included both illicit (e.g. heroin) and prescription medication (e.g. Tramadol). Following data stratification two SNPs: rs118129027 and rs74477937 on chromosome 7 were found to be significantly associated with opioid use disorder. A third SNP rs78707086 did not achieve GWAS significance levels however, it was considered as a potential SNP as it was in strong LD with the top two SNPs (rs118129027 and rs74477937).

Just below the level of significance for GWA studies set at 5.00 × 10^− 8^, seven SNPs on chromosome 2 suggested a potential locus that was associated with opioid use disorder (highlighted in Fig. 1) and considered worthy of further analysis. Five of the SNPs mapped to the *KIAA1211L* gene as illustrated in the regional Manhattan plot of chromosome 2q11.2 (Fig. 2). The SNP rs10175560 was used as the index SNP and was shown to be in high LD with other SNPs mapped to *KIAA1211L*. This could be a possible candidate locus however, the function of *KIAA1211L* is not yet known. Nevertheless, an association between *KIAA1211L* was reported in a study involving bipolar disorder [31]. In a study by Castellanie and colleagues (2014) [32] studied copy number variants (CNVs) in six monozygotic twin pairs for differences in schizophrenia. The monozygotic twins discordant (MZD) approach has been successful in identifying rare variants in schizophrenia. The CNV result in, loss (deletion) or gain (duplication/amplification) of a particular segment of the genome. Castellanie et al (2014) [32] reported CNV loss in *KIAA1211L* in the monozygotic twin pair to be unique in the affected pair with paranoid schizophrenia and not reported in the normal twin pair. This was the first report of this CNV in the database of genomic variants (DGV). Consequently, this CNV loss suggests that this region carries a potential candidate for schizophrenia. In addition, Hicks et al (2016) [33] investigated molecular markers in children with acute lymphoblastic leukaemia (ALL) Central Nervous System (CNS). The current treatment strategy for ALL is a combined systemic chemotherapy and CNS-directed treatment (cranial radiation, intrathecal methotrexate, or combination). The CNS classification is important in ALL patients in order to determine the efficiency of CNS-directed the therapy. The current classification is based on the presence of blast cells in the cerebrospinal fluid (CSF). It is classified to CNS1 (no detectable blast cells), CNS2 (presence of < 5 leukocytes per μl with detectable blast) and CNS3 (presence of > 5 over CNS leukaemia with identifiable blast) [34]. In order to avoid over-treatment or under-treatment in children with ALL, there is a demand for precision assessment of CNS disorders from those without by identifying molecular markers that distinguish each class of CNS. Hicks et al (2016) [33] investigated the differences between gene expression of each level of the CNS in patients. They reported 40 highly significant genes expressed differently between patients with CNS2 and CNS3. The *KIAA1211L* was identified as one of the significant highly expressed genes (4.25 X 10^− 4^) in patients with CNS3 [33]. This can help in determining the intensity of the CNS-directed therapy. Therefore, *KIAA1211L* can be flagged as distinguishing molecular markers for CNS3 typed patients with ALL. The overall finding highlights the involvement of *KIAA1211L* in psychiatric disorders or within the central nervous system and adds weight in support of our results that suggest an association between *KIAA1211L* and opioid use disorder.

In this study, we also investigated SNPs that were in LD with the index SNP rs10175560. The haplotype compositions and their respective frequencies are illustrated in Fig. 3. In block 1, two haplotypes (GAGCG and AGTTA) were significantly associated with opioid use disorder. For haplotype 1 GAGCG the frequency in controls (0.713) was higher than in the cases (0.535), suggesting a possible protective role to opioid use (Fig. 4). On the other hand, haplotype 2 AGTTA was higher in cases (0.369) when compared to the control group (0.219) that suggest possible involvement of this haplotype in the development of opioid use disorder. The remaining three haplotypes that were characterized (GATTA, GAGCA and AGGTA) were not statistically significant with opioid use (Fig. 4).

By expanding the haplotype analysis to include SNPs on chromosome 2 from the *p*-values from 1 × 10^− 8^ to 1 × 10^− 4^, six blocks were generated and illustrated in Additional file 1: Figure. S1. The haplotype variations in each block with the classification between cases and controls are summarised in Additional file 2: Table S1. Block 1 and block 2 mapped to the *KIAA1211L* gene and suggests a potential role in opioid use disorder. In Block 1, two haplotypes, namely CTAAGT (*p*-value = 5.51 × 10^− 8^) and TCTGAC (*p*-value = 2.19 × 10^− 7^) were significantly associated with opioid use. The 2 SNP block 3 mapped to the *ACOXL* (acyl-CoA oxidese like) gene with the GA combination (*p*-value = 7.69 X10^− 7^) shown to be the most significant haplotype. Blocks 4 and 5 mapped to the *VWC2L* gene (von Willebrand factor C domain containing protein 2 like) and block 6 mapped to *OR6B3* (olfactory receptor family 6 subfamily B member 3). There were no direct associations found in the literature between these genes and SUD or specifically opioids. Nevertheless, further investigation would be required to understand the associations reported here and possible mechanisms that may link these genes to opioid use.

Various studies [3, 18, 35–37] have discussed the importance of the q-arm region on chromosome 2 in SUD and other psychiatric disorders (Fig. 5). The *NCK2* gene is located on the long arm of chromosome 2 (2q12.2) is located proximally to the region of the *KIAA1211L* locus identified in this study. Liu et al (2012) [18] reported a significant association between a SNP (rs2377339) on *NCK2* (*p*-value = 3.12 × 10^− 8^) with opioid use disorder in a population of African descent. The *NCK* family is classified as a group of adaptor proteins that interact with other proteins. Specifically, it is involved in the regulation of receptor protein tyrosine kinase signalling and the regulation of actin cytoskeleton and cell movement [32]. These findings suggest evidence for the involvement of the *NCK2* in the pathway of opioid use disorder, that highlight the potential role of the *KIAA1211L* locus in the disorder.

Another genetic region in close proximity to *KIAA1211L* contains the Diazepam Binding Inhibitor (*DBI*). The *DBI* loci on chromosome 2q.14.2 encodes for a protein that has been suggested to be involved in the regulation of number of functions in the CNS, including responses to stress, depression, anxiety and neuropsychiatric disorder [35]. Based on the chromosomal position and the proximity of the *DBI* to the *KIAA1211L,* the possible involvement of the *KIAA1211L* in the CNS should be explored.

Another region of interest on chromosome 2 was mapped to a region bound by 2p14 and 2q14.3. This region has been studied in the context of various behavioural conditions, alcohol dependence, suicide attempts and conduct disorder have been implicated [36]. Dick et al (2010) [36] provided evidence for the involvement of 23 genes (*NTSR2, TRIB2, PPM1G, MEM01, HAAO, MTIF2, CCDC139, EHBP1, BUB1, TTL, CKAP2L, MGAT5, ARHGAP15, KIAA1189, COBLLI, FAM130A2, LPR2, CHN1, PRKRA, PPP1R1C, LOC402117, IL8RA, FARP2*) on chromosome 2 in alcohol dependence. These genes potentially contribute to alcohol dependence, conduct disorder and/or suicide attempts. Other regions across chromosome 2 have been significantly associated with a range of psychiatric disorders. Riley et al (2015) [37] reported genomic imbalance for the region between 2q11.2 and 2q13 in patients with selected clinical symptoms including developmental delay, intellectual disability and congenital anomalies. A deletion in the region from 2q12.2 to 2q13 has been reported in patients with developmental delay and dysmorphic features [38]. Furthermore, a deletion in the 2q21 region was reported in patients with developmental delay/intellectual disabilities, attention deficit hyperactivity disorder, epilepsy and other neurobehavioral abnormalities [39]. The overlap of the regions between studies implicate gene(s) with the CNS that could potential contribute to the disorder.

## Conclusion

Overall, findings in this study propose *KIAA1211L* as a putative candidate locus associated with opioid use disorder. Even though, the function is not fully understood, the region contains genes that are involved in the expression and function of the CNS proteins. In addition, the evidence presented here supports the role of this region in psychiatric disorders, including SUD. Future studies should consider replication of the *KIAA1211L* locus in a larger cohort, including groups of individuals of Arabian descent. In addition, more research is required to investigate the role of *KIAA1211L* in opioid use disorder and other substances. Further assessment of haplotype variations, specifically with reference to opioid use is required in order to understand their roles in opioid use disorder. Future research should consider in depth sequencing of the *KIAA1211L* gene and the surrounding region, comparing variants in opioid use patients and controls to reveal any further genetic associations in order to contribute to the understanding of underlining mechanisms that result in the disorder.

## Supplementary information

**Additional file 1 Figure. S1.** The state of the LD of the SNPs located between the GWAS line and suggestive line (1 X 10 ^− 8^ -1 X 10 ^− 4^) on chromosome 2, based on opioid use disorder patients compared to controls from the UAE population. Block 1, 2 mapped to the *KIAA1211L* gene and is significantly associated with opioid use disorder in this cohort.

**Additional file 2 Table S1.** The distribution of haplotype association between the GWAS line and suggestive line (1 X 10 ^− 8^ -1 X 10 ^− 4^) on chromosome 2 based on opioid users’ patients compared to controls from the UAE population.

## Data Availability

The datasets used and analysed during the current study are available from the corresponding author on reasonable request.

## Abbreviations

ALL: Acute Lymphoblastic Leukaemia
CNS: Central Nervous System
DBI: Diazepam Binding Inhibitor
DSM-5: Diagnostic and Statistical Manual_5
EFR: Emirates Family Registry
GWAS: Genome Wide Association Study
HWE: Hardy Weinberg Equilibrium
IBD: Identity by Descent
MAF: Minor Allele Frequency
MDS: Multi-dimensional Scaling
NRC: National Rehabilitation Centre
QC: Quality Control
QQ: Quantile-Quantile
SUD: Substance Use Disorder
UAE: United Arab Emirates
