# Supplementary material for: Stratified analyses of genome wide association study data reveal haplotypes for a candidate gene on chromosome 2 (KIAA1211L) is associated with opioid use in patients of Arabian descent
Source: BMC Psychiatry. 2020 Jan 31;20:41. doi: 10.1186/s12888-019-2425-8 (PMC6995052; doi:10.1186/s12888-019-2425-8)

Figure S1: The state of the LD of the SNPs located between the GWAS line and suggestive line (1 X 10 $^{-8}$ -1 X 10 $^{-4}$) on chromosome 2, based on opioid use disorder patients compared to controls from the UAE population. Block 1, 2 mapped to the *KIAA1211L* gene and is significantly associated with opioid use disorder in this cohort.

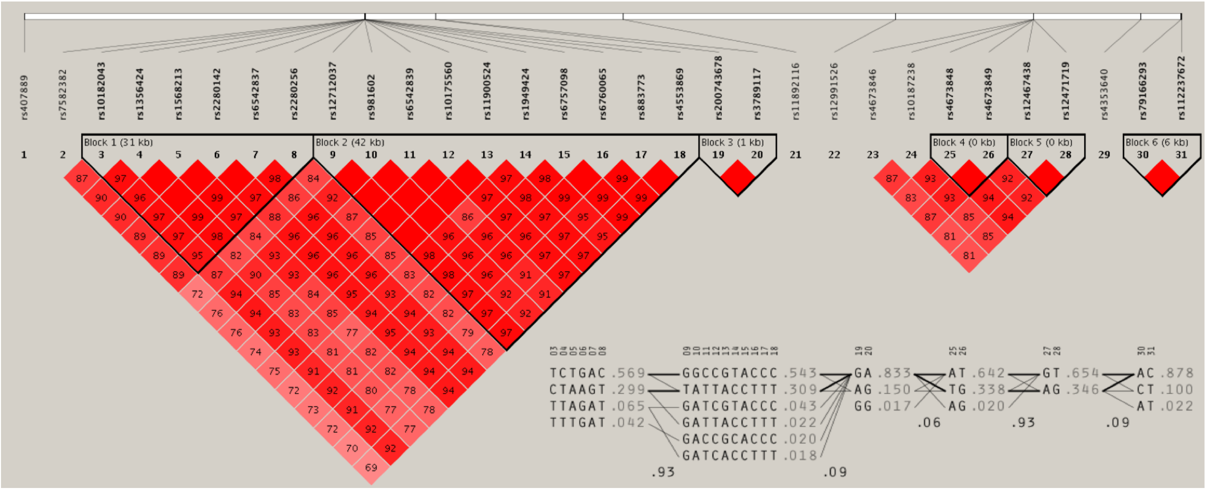

Supplement: Supplementary file 1 — Additional file 1 Figure. S1. The state of the LD of the SNPs located between the GWAS line and suggestive line (1 X 10 − 8 -1 X 10 − 4) on chromosome 2, based on opioid use disorder patients compared to controls from the UAE population. Block 1, 2 mapped to the KIAA1211L gene and is significantly associated with opioid use disorder in this cohort. [file 12888_2019_2425_MOESM1_ESM.pdf]
