# Supplementary material for: Stratified analyses of genome wide association study data reveal haplotypes for a candidate gene on chromosome 2 (KIAA1211L) is associated with opioid use in patients of Arabian descent
Source: BMC Psychiatry. 2020 Jan 31;20:41. doi: 10.1186/s12888-019-2425-8 (PMC6995052; doi:10.1186/s12888-019-2425-8)
Supplement: Supplementary file 2 — Additional file 2 Table S1. The distribution of haplotype association between the GWAS line and suggestive line (1 X 10 − 8 -1 X 10 − 4) on chromosome 2 based on opioid users’ patients compared to controls from the UAE population. [file 12888_2019_2425_MOESM2_ESM.pdf]

Table S1: The distribution of haplotype association between the GWAS line and suggestive line (1 X 10 $^{-8}$ -1 X 10 $^{-4}$) on chromosome 2 based on opioid users' patients compared to controls from the UAE population.

Blocks	Haplotype Frequency	Case, Control Frequencies	Chi Square	P value
**Block 1**				
**TCTGAC**	0.569	0.472, 0.644	26.856	**2.19 X 10 $^{-7}$**
**CTAAGT**	0.299	0.392, 0.225	29.53	**5.51 X 10 $^{-8}$**
**TTAGAT**	0.065	0.053, 0.075	1.843	0.1746
**TTTGAT**	0.042	0.053, 0.034	2.03	0.1542
**Block 2**				
**GGCCGTACCC**	0.543	0.455, 0.614	22.724	**1.87 X 10 $^{-6}$**
**TATTACCTTT**	0.309	0.395, 0.243	24.114	**9.08 X 10 $^{-7}$**
**GATCGTACCC**	0.043	0.034, 0.050	1.349	0.2454
**GATTACCTTT**	0.022	0.038, 0.010	8.257	0.0041
**GACCGCACCC**	0.02	0.021, 0.020	0.006	0.9408
**GATCACCTTT**	0.018	0.013, 0.022	1.064	0.3024
**Block 3**				
**GA**	0.833	0.764, 0.887	24.434	**7.69 X 10 $^{-7}$**
**AG**	0.15	0.204, 0.109	15.672	**7.53 X 10 $^{-5}$**
**GG**	0.017	0.033, 0.004	11.254	**8.00 X 10 $^{-4}$**
**Block 4**				
**AT**	0.642	0.711, 0.587	14.934	**1.00 X 10 $^{-4}$**
**TG**	0.338	0.259, 0.401	20.172	**7.08 X 10 $^{-6}$**
**AG**	0.02	0.030, 0.012	3.789	0.0516

**Block 5**				
**GT**	0.654	0.724, 0.599	15.329	**9.03 X 10 $^{-5}$**
**AG**	0.346	0.276, 0.401	15.329	**9.03 X 10 $^{-5}$**
**Block 6**				
**AC**	0.878	0.937, 0.832	23.055	**1.57 X 10 $^{-6}$**
**CT**	0.1	0.050, 0.138	19.283	**1.13 X 10 $^{-5}$**
**AT**	0.022	0.013, 0.030	3.004	0.083
